# Visual short-term memory relates to tau and amyloid burdens in preclinical autosomal dominant Alzheimer’s disease

**DOI:** 10.1186/s13195-020-00660-z

**Published:** 2020-08-21

**Authors:** Daniel J. Norton, Mario A. Parra, Reisa A. Sperling, Ana Baena, Edmarie Guzman-Velez, David S. Jin, Nicholas Andrea, Juna Khang, Aaron Schultz, Dorene M. Rentz, Enmanuelle Pardilla-Delgado, Joshua Fuller, Keith Johnson, Eric M. Reiman, Francisco Lopera, Yakeel T. Quiroz

**Affiliations:** 1 Department of Psychiatry, Massachusetts General Hospital, Harvard Medical School, 149 13th Street, Rm 10.014, Boston, MA 02129 USA; 2 Gordon College, Wenham, MA USA; 3 School of Psychological Sciences and Health, University of Strathclyde, Glasgow, UK; 4 Autonomous University of the Caribbean, Barranquilla, Colombia; 5 Athinoula A. Martinos Center for Biomedical Imaging, Charlestown, MA USA; 6 Brigham and Women’s Hospital, Harvard Medical School, Boston, MA USA; 7 Grupo de Neurociencias, Universidad de Antioquia, Medellin, Antioquia Colombia; 8 Banner Alzheimer’s Institute, Phoenix, AZ USA; 9 Departments of Psychiatry and Neurology, Massachusetts General Hospital, Harvard Medical School, 149 13th Street, Rm 10.014, Boston, MA 02129 USA

**Keywords:** Associative memory, Autosomal dominant Alzheimer’s disease, Presenilin-1, Tau PET, Biomarkers, Binding

## Abstract

**Background:**

Over the past decade, visual short-term memory (VSTM) binding tests have been shown to be one of the most sensitive behavioral indicators of Alzheimer’s disease (AD), especially when they require the binding of multiple features (e.g., color and shape). Recently, it has become possible to directly measure amyloid and tau levels in vivo via positron emission tomography (PET). To this point, these behavioral and neurochemical markers have not been compared in humans with AD or at risk for it.

**Methods:**

In a cross-sectional study, we compared VSTM performance to tau and amyloid concentrations, measured by PET, in individuals certain to develop AD by virtue of their inheritance of the presenilin-1 E280A mutation. These included 21 clinically unimpaired subjects and 7 subjects with early mild cognitive impairment (MCI), as well as 30 family members who were not carriers of the mutation.

**Results:**

We found that VSTM performance correlated strongly with tau in entorhinal cortex and inferior temporal lobe, and also with amyloid when examining asymptomatic carriers only. The condition requiring binding was not preferentially linked to tau—in fact, the non-binding “shape only” condition showed a stronger relationship.

**Conclusions:**

The results confirm VSTM’s status as an early marker of AD pathology and raise interesting questions as to the course of binding-specific versus non-binding aspects of VSTM in early AD.

## Background

Developing ways to detect Alzheimer’s disease (AD) early, preferably during preclinical stages, is of paramount importance, especially in light of recent trial results indicating that anti-amyloid agents are not effective when administered at clinical stages [1]. Several promising biomarkers have emerged that can aid in early detection, including two biochemical signatures drawn from positron emission tomography (PET) imaging, amyloid aggregation, and tau tangle formation [2, 3]. Amyloid aggregation may be one of the earliest observable changes in AD, appearing at elevated levels at least a decade prior to symptoms, yet it has not shown a robust relation to clinical onset [4, 5]. On the other hand, tau PET imaging has recently come to the fore as a particularly promising biomarker that appears to coincide more closely in time to the earliest observable clinical and cognitive changes in the disease [6, 7] .

Unfortunately, these forms of PET imaging are not widely available and are very expensive even for research trials. This highlights the need to also develop less expensive and more widely distributable screening tools for individuals at risk for AD [8]. These screens can rely on recent developments in the field of cognitive testing where we have witnessed a growth of methodologies showing promise for the preclinical assessment of AD [9]. For novel tools to become reliable screeners, it will be necessary to demonstrate that they can inform about underlying AD pathology. A recent study involving memory markers from the family of selective reminding tests (i.e., the Memory Capacity Test) demonstrated that this is an achievable target [10]. Having such screeners available will greatly increase the purity of samples in clinical trials, and can be disseminated to aging clinics or primary care offices, allowing treatment teams to flag individuals who warrant further diagnostic testing via costly techniques such as PET imaging.

One cognitive marker recently flagged by consensus papers is the Visual Short-Term Memory (VSTM) Binding Test [9, 11–13]. The VSTM Binding Test has been shown to be sensitive to early AD. Binding refers to the ability to integrate object’s features in VSTM to temporarily hold their identity [14, 15]. VSTM binding has been shown to be impaired in aging individuals who have subjective cognitive concerns and those with mild cognitive impairment (MCI) [16]. In addition, individuals in the preclinical stage of AD, who do not yet manifest clinical symptoms, show impaired performance on VSTM Binding Tests, even when they are still able to perform normally on standard neuropsychological tests [17]. Notwithstanding the appeal of the VSTM Binding Test as a potential screening tool for AD, a key question remains in the effort to determine its utility for such a purpose. That is, the degree to which VSTM binding is associated with early disease progression, as measured by key neurochemical markers such as tau and amyloid concentrations, is yet to be demonstrated. So, it is critical to be able to compare amyloid levels to VSTM performance in individuals with preclinical AD.

In the present study, we examined VSTM binding and PET imaging markers in individuals with autosomal dominant Alzheimer’s disease (ADAD) due to the *presenilin-1* E280A (*PSEN1*) mutation. Studying these individuals allowed the examination of VSTM-binding performance, as well as disease biomarkers, in individuals who are still clinically unaffected, or who are in the very early stages of MCI, but who are known with certainty to be in the preclinical stage of AD. The present study is the first to compare VSTM binding performance and tau and amyloid PET imaging data in this or any other population.

We anticipated that VSTM performance would relate to PET markers of brain pathology and that these relations would be especially strong within the color-shape binding condition. Whether VSTM binding performance would relate to tau, amyloid, or both could be anticipated based on the extant literature. For instance, Fleisher and colleagues [18] showed that the growth of amyloid deposits in carriers of the E280A-PSEN1 mutation begins to plateau at around 35 years of age. That is the same age at which Parra and colleagues [17] first reported VSTM Binding deficits as the only cognitive impairment found in these mutations carriers, raising the question of whether amyloid saturation causes, directly or indirectly, VSTM binding deficits. Yet, the temporal coincidence at the group level could be a result of any number of third variables such as the fact that tau accumulation also begins around this age. In addition, other studies have failed to show a strong link between amyloid aggregation and onset of clinical symptoms [4, 5]. On the other hand, given the hypothesized link between tau and neurodegeneration [19], and the recent demonstration of strong relationships between tau and neuropsychological memory tests in preclinical ADAD [7], we might anticipate that VSTM binding would relate most closely to tau in entorhinal cortex (ET) and inferior temporal lobe (IT), given these areas’ importance for memory and their status as representative brain areas for tau accumulation in early Braak stages [20]. Yet, if VSTM is an exceptionally early marker, variance in performance on it might predate tau burden and reach a floor by the time the levels of tau in the brain begin to rise. We hoped the results would provide evidence to clarify which of these competing scenarios is most likely. In addition, we hypothesized that conditions requiring color-shape binding would be most closely related to all biomarkers.

## Methods

### Participants

Participants were 58 individuals from the largest known kindred of individuals with high prevalence of the *PSEN1* E280A mutation; 28 carriers of the mutation, and 30 non-carriers (NC). They were recruited from the Colombian Alzheimer’s Prevention Initiative registry, which currently includes over 3900 living members from the *PSEN1* E280A kindred. Of the carriers, 21 were cognitively unimpaired (unimpaired carriers: UC), and 7 were early MCI, rated by an expert physician trained in neurological assessment and blind to the status of the subjects and purpose of the study (as in previous studies with this kindred). The early MCI carriers were included in correlational analyses to view behavioral performance against biomarker data across a continuum of early disease stages. In this kindred, the median age of onset of MCI is 44 years, with a confidence interval of ± 2 years, while the median age of onset of dementia is 49 years, also with a ± 2-year 95% confidence interval [21]. Participants living in the metropolitan area of the Aburra Valley in Colombia, within a radius of 105 miles of the University of Antioquia, were invited to participate in the study.

### Background assessment

A battery of neuropsychological tests was also administered, as well as a neurological examination, which was performed by a neurologist or a physician trained in assessment of neurodegenerative disorders. The battery included the Mini Mental State Exam (MMSE) and the world list learning test from the Consortium to Establish a Registry of Alzheimer’s disease neuropsychological battery (CERAD).

### VSTM Binding Test

**Fig. 1:**
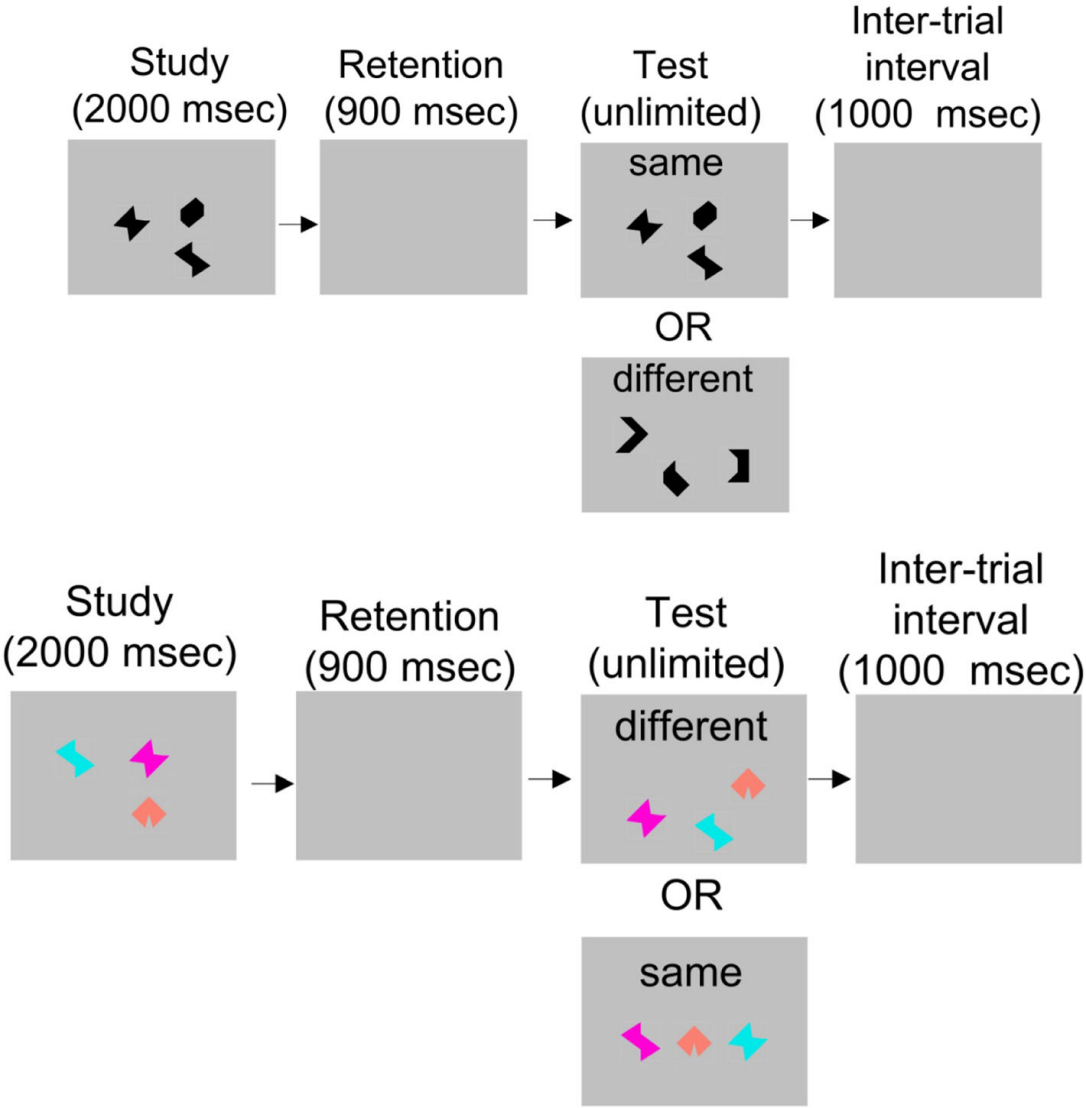
The task was to indicate whether a change in any of the three polygons occurred between the first and second presentations. In all trials, the position of each polygon was different in the first and second presentations. There were two conditions, each presented in its own block of 32 trials. In the shape only (SO) condition, the polygons only varied in shape in both presentations (all were presented as black, on a gray background). In 50% of the trials, the shape of two polygons differed in the second presentation from the first. In the other 50% of the trials, the shapes of all three were identical in the first and second presentations. In the color-shape binding (CSB) condition, each polygon had a different color in the first presentation. In 50% of the trials, on the second presentation, two polygons swapped their colors. On the other 50% of the trials, the same color-shape combinations were presented in both presentations. In both conditions, the first presentation lasted for 2 s, was followed by a blank screen for 900 ms, and then the second set of polygons was presented until the participant responded

The *VSTM Binding Test* uses a change detection paradigm, and it was the same previously used in studies with this kindred and other populations (except for the color only condition which was removed from the paradigm) [17, 22]. In each trial, a trio of polygons was presented initially for comparison with another set, presented later (Fig. 1, see legend for more details on task).

There were two conditions, shape only (SO), which required the recognition of only shapes, and color-shape binding (CSB) which required the recognition of shapes, colors, and which colors went with which shapes (i.e., color-shape binding). Participants were given the choice of responding by a button press or having an experimenter pressing the keys for them, with separate keys indicating “same” or “different.” This was done to mitigate the need for subjects who were not familiar with computers to use extra cognitive resources to remember the button pressing scheme. A condition used to screen for perceptual binding impairments was first administered. Subjects were shown the two polygon sets simultaneously, one at the top and one at the bottom of the screen. There were 10 trials in this condition, and subjects had to score at least 80% accuracy in order to continue with the task. The SO and CSB conditions were then presented in a counterbalanced order. The primary outcome of performance on the VSTM Binding Test was A’, a signal-detection theory based metric, as used in previous studies [17, 23]. In addition to conforming to the convention set by these previous studies, A’ is advantageous as an outcome measure compared with accuracy because it fits better with a signal detection framework in this change detection task. It is also more accurate than the signal detection metric d’ in cases where the RoC curve does not have a slope of one [24].

### Brain image acquisition and analysis

PET imaging was used to acquire measures of amyloid burden and tau binding in the brain. All PET images were acquired using a Siemens/CTI ECAT HR+ scanner (Knoxville TN), which was operated using the following settings: 3D mode, 63 image planes; 15.2 cm axial field of view; 5.6 mm transaxial resolution; and a 2.4-mm slice interval. To measure amyloid, we used 11C Pittsburgh compound B (PiB), which was prepared as described previously [25]. To measure tau, we used the tracer 18F flortaucipir (FTP), which was acquired from 80 to 100 min after injection, using a 9–11-mCi bolus injection in 4 × 5 min frames. PET images were reconstructed and corrected for attenuation, and each frame was evaluated to verify adequate count statistics and the absence of head motion. For PiB, the main outcome measure was mean cortical amyloid, and for tau, we used three regionally defined outcome measures. The areas of interest followed Schultz and colleagues [26] and comprised entorhinal cortex (ER), chosen for its status as the first location of tau buildup, and inferior temporal cortex IT, chosen because it represents the best proxy of early tau spreading to neocortex [3]. In this kindred, inferior parietal cortex has been shown to have a similar temporal course to IT and was therefore not included as a third region [7, 26].

MRI was acquired on a 3T Siemens Tim Trio scanner and included a magnetization = -prepared rapid gradient-echo (MPRAGE) processed with Freesurfer as described previously, to identify gray, white matter and pial surfaces to permit the following region of interest (ROI) parcellations: cerebellar gray (used to calculate SUVR), ER, and IT.

FTP and PiB binding was examined spatial by rigidly coregistering the PET data to each subject’s MPRAGE image using SPM8 (Wellcome Department of Cognitive Neurology, Functional Imaging Laborator, London). The cortical ribbon and subcortical ROIs were transformed into PET native space, and PET data were sampled within each ROI in each hemisphere. The values used for comparison with task performance data were standardized uptake value ratios (SUVR) using cerebellar gray matter, as defined in Freesurfer, as a constant for comparison, and were not partial volume corrected, as previously reported [3]. For PiB, the values used for comparison were the distribution volume ratio (DVR) using cerebellar gray again as a reference tissue. 11C PiB retention was assessed using a large aggregate cortical ROI, which included frontal, lateral temporal, and retrosplenial cortices (FLR) as described previously [27, 28].

18F FTP SUVR and 11C PiB FLR values in mutation carriers and non-carriers were quantified using FS-defined ROIs. PET ROI measures in each group were correlated with age using Pearson’s r. 11C PiB FLR was used as a continuous measure of Aβ and as a categorical variable, with Aβ positivity defined as FLR DVR ≥ 1.2 and Aβ negativity defined as FLR DVR < 1.2 [29].

### General procedures

All participants completed the VSTM Binding Test and were administered magnetic resonance (MRI) and PET imaging at Massachusetts General Hospital in Boston. A battery of neuropsychological tests was also administered, as well as a neurological examination. The battery of neuropsychological tests was administered by a trained lab technician, and clinical examination was carried out by a neurologist. Clinical data were recorded on the Systematized Information System for the Neuroscience Group of Antioquia. All imaging, as well as the VSTM Binding Test, was done within a 1-week span, with three exceptions. One subject was unable to do the PiB scan on the same week as the other testing and was flown back to Boston 3 months later to complete it. Two other subjects did not complete the VSTM Binding Test and were re-tested on the complete test back in Colombia within six months, after the PET testing.

## Results

### Missing data

Two non-carriers were missing data from the SO condition, due to running out of testing time, and were excluded. One non-carrier, two unimpaired carriers, and one carrier with MCI scored below 80% accuracy on the perception condition of the VSTM test and were thus excluded. The final sample sizes for the analyses below were therefore 27 non-carriers, 19 unimpaired carriers, and 6 MCI carriers.

### Demographics

Demographic and clinical information on the sample can be found in Table 1. Asymptomatic carriers did not differ from controls with respect to age, education, gender distribution, MMSE, or other neuropsychological tests.

**Table 1:** Demographics

	MCI	Unimpaired carriers	Non-carriers	*Cohen’s d*, p*
	(*n* = 6)	(*n* = 19)	(*n* = 27)	
Age	44.8 (1.4)	37.5 (6.5)	37.0 (6.5)	.07, .80
Sex	5 F, 1 M	10 F, 11 M	17 F, 11 M	*p* = .43
Education	9.3 (3.4)	11.4 (3.8)	9.9 (4.1)	− 0.37, .22
MMSE	25.5 (3.8)	28.6 (.84)	29.0 (0.94)	.16, .43
Word recall (delayed)	3.5 (2.9)	6.8 (2.1)	7.5 (1.2)	.42, .17

*Based on *t* test comparing preclinical carriers and non-carriers or chi-square in the case of sex

### Biomarker group differences

Unimpaired carriers as a group showed elevated PiB values (mean = 1.31, SD = .17), which differed significantly from non-carriers, *t*(18.8) = 6.76, *p* < .001, whose mean was 1.04, SD = .03). Using a standard cutoff of PiB ≥ 1.20, 15 of the 21 unimpaired carriers (plus the 7 with MCI) were classified as amyloid positive, while 6 were amyloid negative. Unimpaired carriers showed elevated tau levels compared to non-carriers in both areas of interest—ET, *t*(23.7) = 5.22, *p* < .001, IT, *t*(29.1) = 2.66, *p* = .01. In sum, both amyloid and tau were elevated in unimpaired PSEN1 carriers.

### VSTM performance

The order in which the conditions were administered did not significantly affect performance. Those who did the CSB condition first had non-significantly higher A’ scores for the SO condition, *t*(50) = .54, *p* = .59, *d* = .15, and also for the CSB condition, *t*(50) = .75, *p* = .46, *d* = .21.

Performance in unimpaired carriers and non-carriers was compared using mixed design ANOVA, with condition as the within-subject factor and genetic status as the between-subject grouping variable. This analysis showed a main effect for condition, *F*(1, 44) = 62.35, *p* < .001, *η*^2^ = .59, as well as for group, *F*(1, 44) = 5.02, *p* = .03, *η*^2^ = .10, but no interaction between the two, *F*(1, 44) = 1.13, *p* = .29, *η*^2^ = .03. Including the MCI subjects along with the unimpaired carriers strengthened the main effect of group, *F*(1, 49) = 5.48, *p* = .007, *η*^2^ = .18, but did not change the lack of interaction between group and condition, *F*(1, 49) = 1.45, *p* = .25, *η*^2^ = .06. In sum, VSTM performance was hampered in unimpaired PSEN1 mutation carriers. The difference was not specific to CSB condition, as it was in a previous study [17].

### VSTM as a function of age

**Fig. 2:**
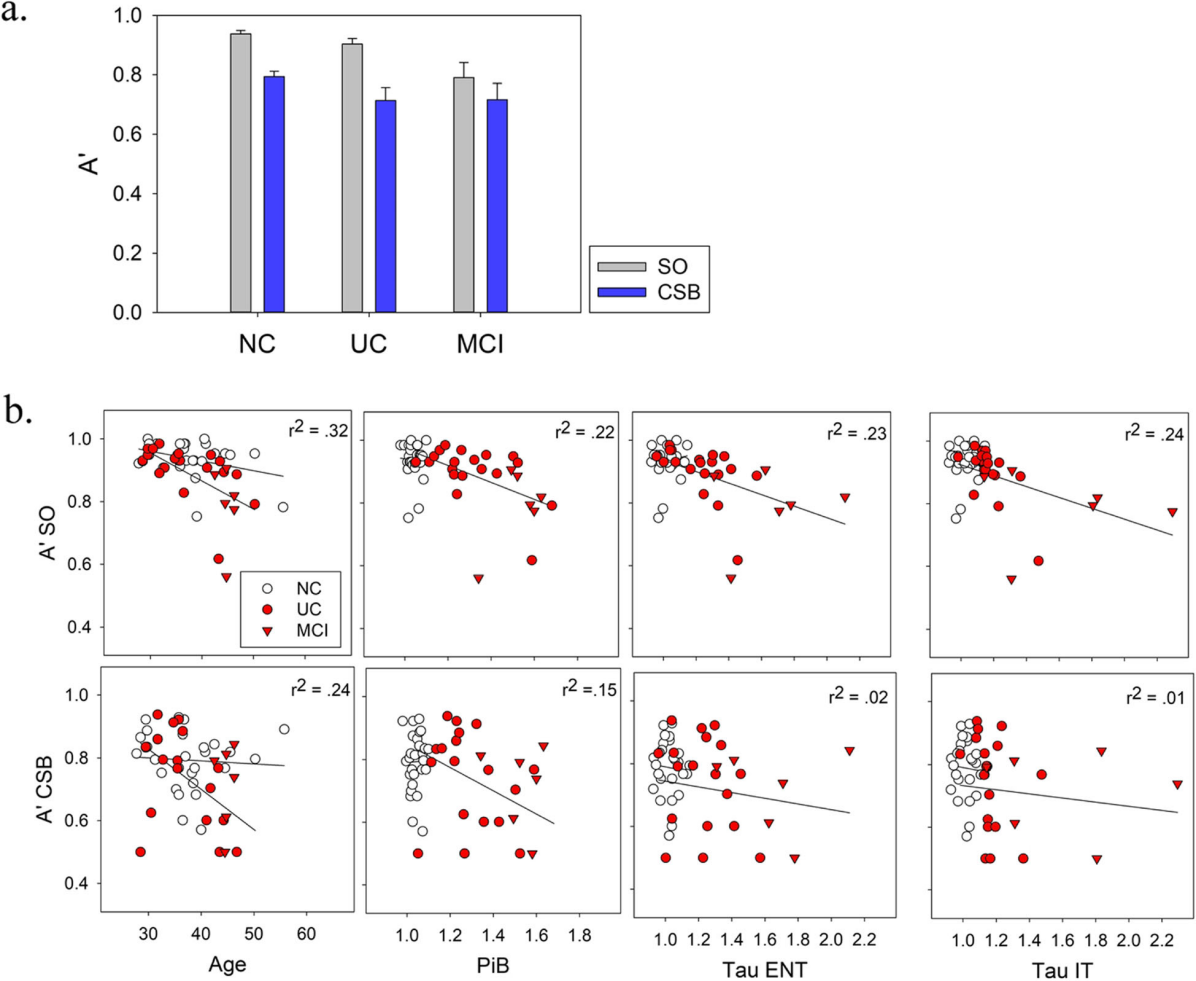
**a** Group means for A’ in the shape only (SO) and color-shape binding (CSB) conditions. Error bars denote standard error. NC, non-carrier; UC, unimpaired carrier; MCI, carrier with mild cognitive impairment. **b** Scatterplots of visual short-term memory performance as a function of age and biomarker levels. In this group of carriers, age is tantamount to proximity to disease onset minus a constant. *r*^2^ values refer to the entire carrier group, combining MCI and UC

In this kindred, where the median age of MCI onset is 44 years, age is equivalent to proximity to expected clinical onset, plus a constant of 44. Therefore, we examined the progression of VSTM performance as a function of age in carriers to see if it progressed linearly with proximity to expected clinical onset. As shown in Fig. 2, A’ decreased significantly with age in carriers in the SO condition, *r*(23) = − .57, *p* = .003, as well as the CSB condition, *r*(23) = − .49, *p* = .01. Controlling for education, both correlations remained significant—SO: *r*(22) = − .51, *p* = .01, CSB: *r*(22) = − .43, *p* = .04. In non-carriers, there was not a significant relationship with age and VSTM performance in the SO condition, *r*(25) = − .35, *p* = .07, or the CSB condition, *r*(25) = − .07, *p* = .74.

### Comparison of VSTM performance with PET biomarker levels and other factors

We examined the relation between PET biomarkers and VSTM performance in both carriers and non-carriers, as shown in Fig. 2. For non-carriers, A’ in the SO and CSB conditions were not correlated with PiB, ER tau, or IT tau, −.24 < *r*(25) < .04, *p* > .2. For carriers, correlation coefficients comparing VSTM performance and PET biomarkers are shown in Table 2. Analogous results for the group of asymptomatic carriers separately in Table 3. Examining the entire group of carriers for relations between VSTM performance neuropsychological measures, we found the following.

**Table 2:** Correlations between VSTM performance and biomarkers in all carriers (*n* = 25)

	PIB	ER tau	IT tau
SO	*r* = − .47	*r* = − .48	*r* = − .49
	(*p* = .02)	(*p* = .02)	(*p* = .01)
CSB	*r* = − .39	*r* = − .14	*r* = − .11
	(*p* = .06)	(*p* = .50)	(*p* = .60)

**Table 3:** Correlations between VSTM performance and biomarkers in all asymptomatic carriers only (*n* = 19)

	PIB	ER tau	IT tau
SO	*r* = − .56	*r* = − .53	*r* = − .72
	(*p* = .01)	(*p* = .02)	(*p* = .001)
CSB	*r* = − .50	*r* = − .26	*r* = − .30
	(*p* = .03)	(*p* = .27)	(*p* = .21)

MMSE scores were not significantly correlated with A’ in the SO condition, *r*(17) = .37, *p* = .12, or the CSB condition, *r*(17) = .24, *p* = .32. Word list learning scores were correlated with A’ in the SO condition, *r*(17) = .53, *p* = .02, but not in the CSB condition, *r*(17) = .37, *p* = .12.

### Classification of biomarker status by VSTM and other variables

**Fig. 3:**
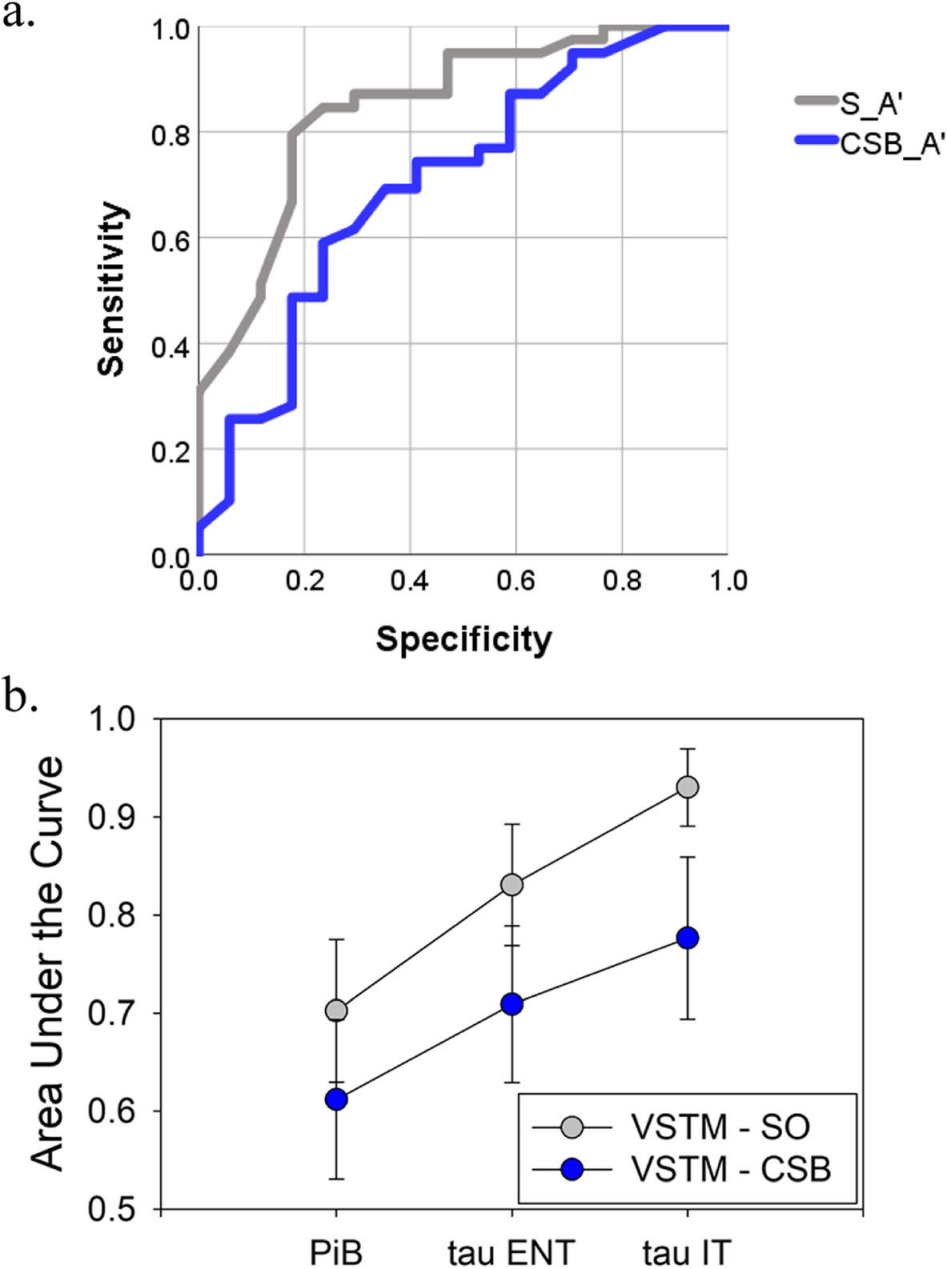
**a** Results from receiver operating curve showing the ability of visual short-term memory performance to classify individuals as biomarker positive. Data are shown for the tau in entorhinal cortex, where biomarker positivity was defined as 3 standard deviations above the non-carrier mean level. According to this system of classification, there were 23 amyloid positive subjects (29 negative), 14 ENT tau positive subjects (38 negative), and 7 IT tau positive subjects (45 negative). Areas under the curve (AUC) are shown for each analysis in **b**. **b** Summary of VSTM performance in ROC analysis across three biomarker types. Means areas under the curve are shown with standard error. Data from the VSTM shape only (SO) condition were superior in classifying individuals as positive for all three biomarkers

Receiver operating curves were used to assess the sensitivity and specificity of VSTM performance for indicating status as possessing elevated levels of amyloid and tau. Results are shown in Fig. 3. All subjects were included in the analysis and grouped as biomarker-positive or biomarker-negative. To generate a cutoff value to make biomarker status binary, we used the standard 1.2 cutoff for PiB and used the value of 3 standard deviations above the NC mean for tau in ENT and IT. Figure 3b shows the area under the curve for the SO and CSB VSTM conditions.

## Discussion

### Summary of results

The present study was the first to compare VSTM performance with in vivo amyloid and tau burdens using PET imaging. It did so in carriers of the PSEN-1 E280A mutation, along a continuum of early AD, both unimpaired and early MCI. VSTM performance correlated strongly with tau aggregation in ER and IT. The relation between tau and VSTM performance persisted when restricting the analysis to the asymptomatic carriers, though it was stronger for the SO condition in that case. Amyloid correlated significantly to CSB and SO VSTM performance when examining the asymptomatic carriers separately from the MCI participants. These results demonstrate a relationship between progressing disease pathology and VSTM performance within the very early stages of AD, before clinical presentation.

### Potential reasons for the preferential relation between VSTM SO and biomarkers

With respect to VSTM relation to biomarkers, we expected to find a preferentially strong relationship between the CSB condition and PET biomarkers of early AD pathology—stronger than the relation between those biomarkers and performance on the SO condition. This did not prove to be the case. The CSB condition correlated with PIB at a similar level as the SO condition but was only significant when restricting the analysis to the asymptomatic carriers. This is consistent with the notion that feature binding is a preclinical marker related to the earliest neuropathological changes caused by the disease. On the other hand, the correlations between tau markers and performance on the SO condition were stronger than those with the CSB condition. Why would this be the case? There are three tentative answers to that question.

First, the brain changes responsible for early CSB impairment in AD may not be especially related to tau buildup in ER or IT, at least levels that are measurable by PET. The ER may be a structure supporting post-unitization memory processes. Once features are integrated through low-level binding functions [30], they become a unit. Previous studies suggest that such unified representations pose no extra cost to memory relative to single feature objects [14, 15]. Taken together this evidence suggests that the ER might be involved in post-unitization processing with downstream structures in the ventral visual pathway (lateral occipital complex of the fusiform gyrus), being more relevant for feature binding. This is in line with a proposal by Staresine and Devachi [31] and other findings [32, 33].

A second explanation has less to do with neuroanatomy and more with the timelines of VSTM impairment and tau spread in preclinical AD. Impairment on the CSB condition, even to the point of performing at chance levels, may emerge before tau is measurable. If this were the case, performance may approach a floor in subjects before they display measurable tau in ER cortex or later areas, removing any relationship between performance and measurable tau. In the SO condition, on the other hand, performance in non-carriers is quite high, and there is more room for a relationship to develop with tau as performance begins to, and continues to deteriorate. The trend of our data suggests this might be a plausible account. Figure 2 (lower panel) shows that the lower level of CSB performance—as compared to SO performance—is less able to be further affected by increases in tau, while SO continues to decline as tau deposits grow. This is similar to other more traditional neuropsychological tests, which are less sensitive to the preclinical stages of AD, but decline reliably with disease progression. It is also similar to a study on VSTM capacity for letters, which declined in individuals with diagnosed AD, but not MCI, meaning this capacity tracked well with later disease progression [34]. This could also explain why increases in amyloid did not further hamper VSTM performance at stages where tau pathology has been triggered. Nevertheless, the results presented here can only be considered preliminary as they were drawn from a small sample comprising both symptomatic and asymptomatic mutation carriers.

The third explanation has to do with testing characteristics. Assuming that there is a genuine deficit in ability in a certain group, a test’s ability to reveal that deficit is partially determined by the difficulty level of the task [35]. The ideal mean performance for the control group in such a task is equidistant from floor and ceiling effects (e.g., an A’ of roughly .75 in the present study). In the present study, performance in both the SO and CSB conditions was slightly lower in both groups than in previous studies. This could be due to lack of practice trials, or fatigue in our subjects, who completed the VSTM task in the midst of a comprehensive battery of tests in our COLBOS study. Regardless of the reason, the lower accuracy overall probably had the effect of moving the CSB accuracy towards a floor effect, and the SO condition away from a ceiling effect into the ideal zone for discriminating. It is likely that this comparatively worse performance made the SO condition more sensitive to reveal any true deficits in the carrier group and the CSB condition less sensitive to do so. This situation may work in combination along with the neuroanatomical and time-course factors mentioned above to produce the present results that favor the sensitivity of the SO condition.

### Integrating VSTM results with previous literature

It has been proposed that deficient performance on the CSB condition is a specific phenotype associated with early AD pathology [22, 36]. While the present results support the idea that the VSTM binding test overall is sensitive to preclinical AD (with the entire carrier group, with or without the MCI subjects included, showing a deficit on the task), the previously demonstrated interaction effect between group and condition was not shown here. This supports the idea that VSTM is one of the earlier behavioral markers of preclinical AD, but leaves open the question of why CSB did not stand alone as the more sensitive differentiator of carriers vs. non-carriers, as it did in past studies. We can think of two potential accounts for this observation, the first being simply that the small sample size required a large effect size to produce a significant result. A second account would derive from the fact that the entire carrier group included patients in the MCI stage.

A previous study showed that in MCI stages, the Group x Condition interaction previously reported in unimpaired cases of ADAD is not present when a version of the test using three items, rather than two, is employed [16]. The present results suggest that assessment of VSTM binding requires careful planning with respect to task difficulty [35], which can be modulated in many ways. For example, using a task that measures performance across multiple memory load conditions (e.g., 2 and 3 items) would more reliably allow tracing the continuum of AD. Parra and colleagues recently investigated this issue [37] and showed that the CSB specificity relative to SO is lost in patients who are in more advanced or in symptomatic stages (i.e., MCI). They argue that this seemingly reflects the combined impact of disease and memory load. When patients were assessed with lower memory load, the typical Group x Condition interaction suggesting CSB specific impairments was restored.

It is noteworthy that although the demographic characteristics of this and previous samples match quite well in terms of age and education, the present results differ in degree from previous VSTM-binding studies in the same kindred. Chiefly, A’ values for both SO and CSB are a bit lower in our sample than in previous studies [22]. It is possible that the generally lower accuracy in the present sample caused the data to approach a floor effect more rapidly and therefore reduced the degree to which the CSB condition could meaningfully covary with the PET biomarkers. There are two potential reasons for this lower accuracy. First, this study took place within a larger, weeklong study of tests which were rather grueling for some subjects, and could have resulted in lower accuracy, although this has not been shown in other tasks and tests using this Boston sample (e.g., [7, 38, 39]). Second, a slight difference in procedure compared with previous studies may be an important factor in the difference between past and present results in terms of color binding performance in preclinical AD. In our procedure, subjects were presented with a diagram of the task, and then began the perceptual condition. In the shape and color-shape conditions, a new diagram was presented and the task re-explained, but no practice trials were done. In the previous studies by Parra and colleagues, there were 15 practice trials in all conditions, which may have improved performance.

Other studies have compared VSTM performance to biological measures of disease progression in early ADAD. A prior study showed that VSTM binding performance decline in AD is linked to white matter integrity [40]. This study showed a correlation between white matter integrity and color-binding performance only in individuals in the clinical stage of the disease. We found that tau accumulation in the ER and IT was associated with performance on the SO condition of the task even in individuals with no clinical diagnosis. This differing pattern across the two studies is consistent with the idea that ER and IT tau emerges prior to the destruction of white matter integrity in the disease, although recently it was shown that structural tract alterations in the hippocampal cingulum bundle predicted later tau accumulation in the cingulate [41].

### Limitations

The present results should be interpreted cautiously due to the small sample size used. Although the sample is relatively large for its kind, comprising 52 members of the Colombian kindred with high *PSEN-1* prevalence, it is still in need of replication. In addition, since our sample spanned a range of preclinical and prodromal stages, including asymptomatic and mild MCI, the latter subgroup’s performance is likely confounded by generalized deficits (which could be further affected by the lower education values in this group as compared to non-carriers). It would be ideal to have a sufficiently powered study to examine the differential deficit that may exist in VSTM over and above these. Importantly, the main findings of the study—correlations between tau biomarkers and VSTM performance—remained robust when the MCI subjects were excluded.

### Future directions

Whether the results will generalize to sporadic AD and normal aging samples is unknown. Longitudinal studies will also reveal the extent to which changes in VSTM performance may be even more informative of one’s tau status than raw performance at one time point, which was all that was available in the present study. Finally, a host of stimulus factors, including presentation time, number of items, and the presence or absence of a mask remain to be explored for the purpose of maximizing the sorts of memory failures that are specific to preclinical AD [37]. Perhaps more important, the present study found the SO condition to be more closely linked to tau levels than the CSB condition in both the RoC analysis and the linear correlations, while prior studies have found that the CSB condition, but not the SO condition, is strongly linked to early disease progression. It is important to disentangle these complex results. Is the lower accuracy in both conditions in the present study as compared to prior studies to blame for moving the CSB accuracy towards a floor effect, and the SO away from a ceiling effect into a more discriminating difficulty level? Or, is the SO condition simply related strongly to tau (which occurs relatively late in the preclinical stage of the disease) while the CSB condition is indeed an early marker that is especially sensitive to preclinical AD despite its lack of relation to tau? These questions remain to be clarified in future studies with larger samples.

## Conclusions

In this cross-sectional study of individuals from the Colombian kindred, VSTM performance correlated to varying degrees with PET measurements of biochemical disease progression. Across the entire carrier group, tau correlated with VSTM performance in the SO condition, but not the CSB condition, and amyloid did not correlate with VSTM performance in the entire carrier group. When restricting analyses to the asymptomatic carriers only, both amyloid and tau correlated with VSTM performance. While both VSTM conditions are promising areas for further study, it will be necessary to continue exploring the precise stimulus conditions that are optimal for serving as a proxy for biochemical pathology in AD or for discriminating between individuals with and without preclinical AD. Future studies should embark on such a systematic exploration.

## Data Availability

Data are not currently publicly available due to the sensitive nature of the rare kindred individuals tested.

## Abbreviations

MCI: Mild cognitive impairment
AD: Alzheimer’s disease
ADAD: Autosomal dominant Alzheimer’s disease
CERAD: Consortium to Establish a Registry of Alzheimer’s disease neuropsychological battery
CSB: Color-shape binding
DVR: Distribution volume ratio
ER: Entorhinal cortex
FLR: Frontal, lateral temporal, and retrosplenial cortices
FTP: 18F flortaucipir
IT: Inferior temporal cortex
MMSE: Mini Mental State Exam
MPRAGE: Magnetization = -prepared rapid gradient-echo
MRI: Magnetic resonance imaging
NC: Non-carriers
PET: Positron emission tomography
PiB: 11C Pittsburgh compound B
*PSEN1*: *Presenilin-1* E280A
ROI: Region of interest
SO: Shape only
SUVR: Standardized uptake value ratios
UC: Unimpaired carriers
VSTM: Visual short-term memory
